# Effects of different nitrogen treatments on the growth and nitrogen metabolism of *Machilus thunbergii* seedlings

**DOI:** 10.3389/fpls.2025.1684502

**Published:** 2025-12-17

**Authors:** Fenghou Shi, Haoyi Feng, Xiaojing Li, Yuhui Zhang, Yanan Bai, Boqiang Tong, Yizeng Lu

**Affiliations:** 1 Collaborative Innovation Centre of Sustainable Forestry in Southern China, College of Forestry and Grassland, Nanjing Forestry University, Nanjing, China; 2 Yudu County Forestry Bureau, Ganzhou, China; 3 Shanghai Forestry General Station, Shanghai, China; 4 Shandong Provincial Center of Forest and Grass Germplasm Resources, Jinan, China

**Keywords:** *Machilus thunbergii*, nitrogen fertilizer, seedling growth, nitrogen metabolism, nitrogen accumulation, nitrogen metabolism enzyme

## Abstract

**Introduction:**

As a native tree species in China, *Machilus thunbergii* is highly responsive to nitrogen fertilization. However, related studies are scarce. This research seeks to elucidate how different nitrogen fertilizers affect its growth and nitrogen metabolism across different growth stages, thereby determining the most suitable type and establishing a scientific foundation for its fertilization.

**Methods:**

This study aimed to investigate the effects of topdressing with different nitrogen fertilizers on the growth and nitrogen metabolism of *M. thunbergii* seedlings, with the goal of providing a scientific basis for optimized nitrogen fertilization management in *M. thunbergii* cultivation. The study used 3-year-old *M. thunbergii* seedlings as the material, and the fertilization rate was 3 g per seedling. The experiment was conducted in a one-way randomized block design with four treatments, including the control treatment and three nitrogen fertilizer treatments: urea (amide nitrogen fertilizer), ammonium sulfate (ammonium nitrogen fertilizer), and sodium nitrate (nitrate nitrogen fertilizer). From the start of the experiment, the branch and leaf morphology, the height growth, and the basal diameter growth of the seedlings in each treatment were monitored periodically. The activities of the nitrogen-metabolizing enzymes, such as nitrate reductase, glutamine synthetase, glutamate synthetase, and glutamate dehydrogenase, in the leaves were also measured. At growth cessation, all treatments were evaluated for biomass production, root morphological characteristics, and total nitrogen content in different plant parts (i.e., roots, stems, and leaves).

**Results:**

Due to the high nitrogen content in the cultivation substrate, the application of the different nitrogen fertilizers induced varying levels of fertilizer injury. Temporal analysis revealed that the growth inhibition was not uniform across stages. While all nitrogen treatments ultimately suppressed the overall height and diameter growth compared with the control, the timing and the intensity of these effects varied. For instance, the urea treatment initially showed less inhibition, whereas the sodium nitrate treatment consistently exhibited the strongest inhibitory effect throughout the experiment. Similarly, the promotion of nitrogen metabolism enzyme activity by the different fertilizers also displayed distinct temporal patterns, with peaks occurring at different measurement points. All nitrogen treatments increased the nitrogen content in the root, stem, and leaf parts, but decreased the nitrogen translocation efficiency of *M. thunbergii* seedlings. All nitrogen treatments increased the nitrogen accumulation in the roots and stems of seedlings. Urea treatment enhanced foliar nitrogen accumulation, whereas both the ammonium sulfate and sodium nitrate treatments reduced foliar nitrogen accumulation.

**Conclusion:**

All three nitrogen treatments significantly influenced both the growth and physiological indices of *M. thunbergii* seedlings. While generally enhancing the nitrogen metabolism and accumulation, improper selection of fertilizer types or excessive application rates elevated the tissue nitrogen concentration, inducing phytotoxic effects that ultimately inhibited seedling growth. In this research, sodium nitrate had the greatest toxic effect on *M. thunbergii* seedlings, followed by ammonium sulfate and urea. Among the nitrogen fertilizers tested, urea proved superior at an application rate of 3 g per plant for 3-year-old *M. thunbergii* seedlings.

## 1 Introduction

As the predominant mineral nutrient for plants, nitrogen is an important component of various secondary metabolites in plants and is involved in the synthesis of proteins, nucleic acids, and phospholipids, among others. It also has significant impact on plant growth and nitrogen metabolism (Farhan et al., 2024; Gong et al., 2024; Langworthy et al., 2023; Xu et al., 2017). Given the naturally low nitrogen availability in the majority of soils, nitrogen fertilization is essential for seedling cultivation. Optimal nitrogen fertilizer application facilitates higher chlorophyll content (Qiu et al., 2024) and nutrient balance (Dong W.N, et al., 2025), thereby promoting overall plant development (Li Y.L, et al., 2023). However, inappropriate application can lead to a series of problems such as low nitrogen fertilizer utilization efficiency, plant growth inhibition, reduced yield, soil compaction, and environmental pollution (Saurav et al., 2024). Since the 1980s, with the objective of enhancing agricultural and forestry yields, the amount of nitrogen fertilizer applied in China has rapidly increased, causing a series of issues. Aiming to avoid unsuitable fertilization, numerous studies have been conducted on the types and the amounts of nitrogen fertilizers. The main types of nitrogen absorbed by plants from the soil are nitrate nitrogen (NO_3_^−^–N) and ammonium nitrogen (NH_4_^+^–N). These distinct forms of nitrogen have significant effects not only on the plant growth but also on its physiology (Yin and Zhang, 2025; Ge et al., 2024). Therefore, studying the response of plants to different forms of nitrogen is of great significance in agricultural and forestry applications. Nitrogen assimilation, the conversion of inorganic nitrogen to organic forms, can be evaluated through the activities of key enzymes [e.g., nitrate reductase (NR), glutamine synthetase (GS), glutamate synthetase (GOGAT), and glutamate dehydrogenase (GDH)] (Yu et al., 2024). The amino acids synthesized in this process are further converted into various substances, such as proteins, chlorophyll, vitamins, and nucleic acids, among others, to support plant life activities (Kusano et al., 2011).

*Machilus thunbergii* is a broad-leaved evergreen tree in the genus *Machilus* of the family Lauraceae, which is widely distributed in the provinces of Shandong, Zhejiang, and Guangdong in China (Huang et al., 2013). This species possesses significant ornamental, economic, and medicinal values, as well as ecological functions including soil/water conservation, salt tolerance, and wind resistance. *M. thunbergii* has broad potential for cultivation and utilization. Current studies on *M. thunbergii* have primarily concentrated on population distribution (Li et al., 2024; Zhang et al., 2023), growth and reproduction (Li et al., 2022; Qian, 2022), and landscape and medicinal value utilization (Ma and Huang, 2019), among others. These studies are of great significance for the protection of *M. thunbergii*. However, research on the fertilization strategies for *M. thunbergii* seedlings is limited, with no established standardized fertilization protocols. Fertilization experiments were conducted on *Lindera megaphylla* and *Cinnamomum chekiangense*, which belong to the family Lauraceae, similarly to *M. thunbergii*. The results showed that, among the elements nitrogen, phosphorus, and potassium, nitrogen had the greatest impact on the growth of seedlings (Dong et al., 2011; Song and Fei, 2013). Therefore, it is of practical significance to study the effects of different nitrogen treatments on the growth and nitrogen metabolism of *M. thunbergii* seedlings. However, the response of plants to nitrogen is dynamic, not static. Therefore, a fertilizer that provides a strong initial growth stimulus may not offer sustained benefits, and the effect on nitrogen metabolism can vary over time. To comprehensively evaluate the effects of different nitrogen forms, it is essential to monitor the key growth and physiological parameters at multiple stages throughout the growth cycle. This time series approach is crucial for the identification of nitrogen sources that provide balanced and persistent advantages.

Employing 3-year-old *M. thunbergii* container seedlings, this study evaluated the effects of different nitrogen types on the seedling growth, nitrogen transport, and assimilation through sequential measurements over 150 days. The aim was to identify the optimal nitrogen fertilizer at the specified application level, thereby establishing a scientific basis for optimal fertilization and facilitating protection. The response of *M. thunbergii* seedlings to nitrogen fertilization exhibits a dynamic nature, with the effects varying by nitrogen form, manifesting as heterogeneous patterns such as early-stage rapid promotion or long-term sustained effects. Therefore, this experiment was conducted with a measurement frequency of every 30 days to monitor the seedling height growth, the ground diameter growth, and the nitrogen metabolism enzyme activity. This approach was designed to obtain continuous multi-time point data, enabling a comprehensive analysis of the impacts of the three nitrogen fertilizers on the seedlings over both the short-term and entire experimental periods, thereby providing substantial and reliable data for subsequent research.

## 2 Materials and methods

### 2.1 Test site and experimental materials

**Figure 1:**
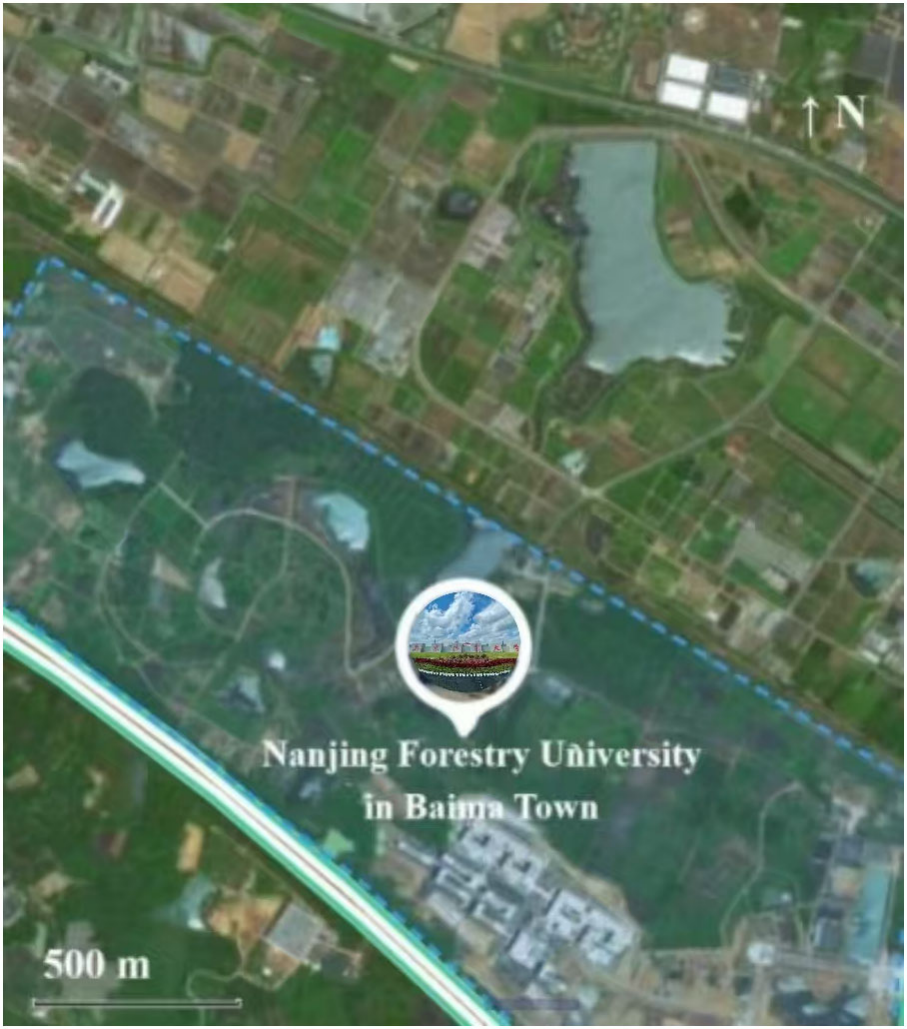
Satellite image of the test site.

The fertilization experiment with *M. thunbergii* seedlings was conducted at the teaching and research base of Nanjing Forestry University in Baima Town (119.181946° E, 31.605082° N, altitude of 10 m). A satellite image is provided in **Figure 1**. The site features a subtropical monsoon climate with favorable hydrothermal conditions: a mean annual temperature of 15.5°C (summer maximum of 38°C and winter minimum of −8°C), an annual precipitation of 1,053 mm, and sunshine duration 2,240 h. We selected 3-year-old *M. thunbergii* seedlings for the experiment. The nursery seeds were obtained from the natural population of *M. thunbergii* in Sheshan Island, Shanghai Municipality. *M. thunbergii* seedlings with nearly uniform growth were transplanted into pots for acclimatization on March 21, 2021 (pot size: height, 20.5 cm; upper aperture, 24.5 cm; lower aperture, 18 cm), one plant per pot. Each pot was filled with 4 kg seedling substrate (bulk density, 0.31 g cm^−3^; total porosity, 65%; pH 6.59; EC, 2.71; organic matter content, 37%; and total nitrogen, phosphorus, and potassium content, 2%), which was produced by Jiangsu Xingnong Substrate Science and Technology Co. Ltd. (Zhenjiang, China). In July, the experimental seedlings were transferred into a simple greenhouse for standard cultivation management during the adaptation period. On April 17, 2022, experimental seedlings (80–90 cm tall, with uniform growth) were selected for the formal trial.

### 2.2 Experimental design

The experiment was conducted using a one-way randomized block design with four treatments: urea (amide nitrogen fertilizer, 46.7% N) treatment, ammonium sulfate (ammonium nitrogen fertilizer, 21.2% N) treatment, sodium nitrate (nitrate nitrogen fertilizer, 16.5% N) treatment, and a control (CK) treatment with no nitrogen fertilizer applied. The experiment employed three replicates per treatment, with 12 seedlings per replicate, giving a final sample size of 144 seedlings. The initial fertilization day was designated as 0 DAT (days after transplantation). Nitrogen was applied at a rate of 3 g per seedling. This dosage was determined based on the Jiangxi Provincial Administration for Market Regulatio, (2018), which recommends an appropriate nitrogen application rate of 2–3 kg per 667 m^2^. Calculated based on the substrate of each pot in this experiment, the application rate was set at 3 g per plant divided equally into three applications (0, 30, and 60 DAT), with each dose dissolved in 200 ml deionized water and applied to the substrate. The entire fertilizer experiment was maintained for 150 days, during which the seedlings were under standard irrigation management throughout. Detailed treatment specifications are presented in **Table 1**.

**Table 1:** Formulation and application rates of nitrogen fertilizer treatments.

Treatment	Nitrogen application (g per seedling)	Nitrogen fertilizer application rate (g per seedling)
Control	0	0
Urea	3	6.49
Ammonium sulfate	3	14.29
Sodium nitrate	3	18.21

All chemicals (i.e., urea, ammonium sulfate, and sodium nitrate) were of analytical grade with a purity of ≥98%.

### 2.3 Observation of the seedling morphology and determination of the growth indicators

The stem and leaf morphology was observed and recorded at 30-day intervals for all treatments throughout the experiment. The seedling height and the basal diameter were measured at 30-day intervals on 12 randomly selected seedlings per treatment. Height was measured using a steel ruler (0.1-cm precision), and diameter was measured with digital calipers (0.01-mm precision) (Chen et al., 2023). The growth increments for both parameters were calculated for each interval. At the end of the experiment, three seedlings per treatment were randomly sampled. These seedlings were extracted from the soil manually, divided into three parts (i.e., roots, stems, and leaves) according to the method described by Li et al. (2022), dried under 105°C until the weight remains constant, and then weighed individually with an electronic balance (0.001 g).

### 2.4 Determination of the seedling root metrics

At the end of the experiment, three seedlings per treatment were randomly selected. The roots were carefully excavated from the substrate, gently rinsed with water, and then scanned using an Epson scanner. The total root length, the root surface area, the root volume, the average root basal diameter, and the total root tip number were analyzed using the WinRHIZO PRO 2007 software (Ren et al., 2023).

### 2.5 Determination of the leaf nitrogen-metabolizing enzyme activity

A total of 12 seedlings were randomly selected for each treatment. Throughout the experiment, one or two intact mature leaves from the middle and the upper two to three layers of the seedlings were taken at 30-day intervals, frozen and stored in an ice box for transport back to the laboratory, and then cut and mixed. Subsequently, the activities of NR, GS, GOGAT, and GDH were measured for each treatment using test kits. The kits for the measurement of the enzyme activities were obtained from Suzhou Keming Biotechnology Co. (Suzhou, China).

### 2.6 Determination of the total nitrogen content and accumulation in each part and the transfer factor of the seedling

To investigate the nitrogen uptake and translocation in plants, the nitrogen content and its accumulation were determined at the end of the experiment, and the transfer factor (TF) was studied. To determine the nitrogen content in each part of the seedlings with the different treatments, the seedlings were divided into three parts: roots, stems, and leaves. Pooled samples from the same treatment and plant part were dried, ground, and digested with sulfuric acid–hydrogen peroxide. The total nitrogen content was determined using the micro-Kjeldahl method, adhering to the established standard for plant NPK determination (Ministry of Agriculture and Rural Affairs of the People’s Republic of China, 2011).

(1) $$NA=N_{c}\times DM$$

where NA is the nitrogen accumulation (in milligrams per seedling), *N*_c_ is the nitrogen content in a specific plant part, and DM is the dry matter of that part.

(2) $$TF=\frac{N_{stem}+N_{leaf}}{N_{root}}$$

where TF is the nitrogen transfer factor, *N*_stem_ is the nitrogen content of the stems, *N*_leaf_ is the nitrogen content of the leaves, and *N*_root_ is the nitrogen content of the roots (Mostafa et al., 2021).


Nitrogen accumulation in each part of the seedling(mg per seedling)=nitrogen content in each part×dry matter of the part.

### 2.7 Data processing and analysis

All statistical analyses were performed using SPSS 23.0. Significant differences were determined using one-way analysis of variance (ANOVA). Statistical significance was defined as *p* < 0.01 and *p* < 0.05. Graphs were generated using Origin 2024 software.

## 3 Results

### 3.1 Assessment of *M. thunbergii s*eedling branch and leaf morphology

**Figure 2:**
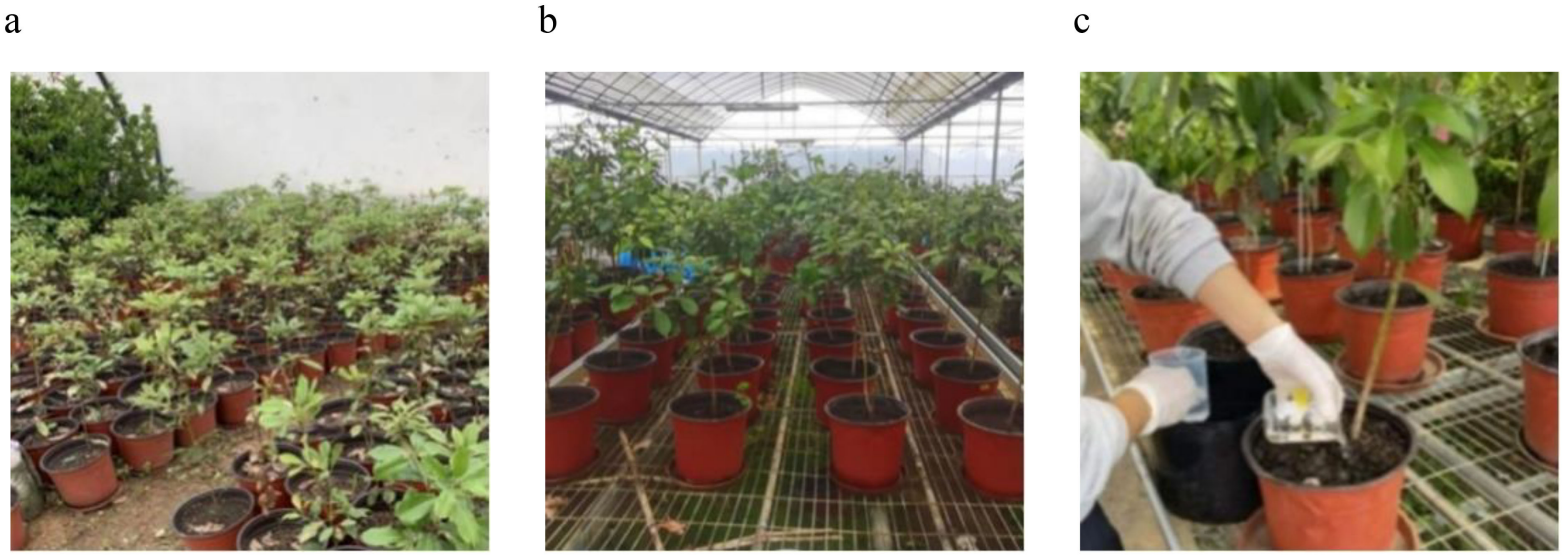
Key stages in the fertilization experiment of *Machilus thunbergii* seedlings. **(A)** Transplantation of seedlings into experimental pots. **(B)** Acclimatization period prior to fertilization. **(C)** Application of the nitrogen fertilizer treatments.

*M. thunbergii* seedlings received distinct nitrogen treatments, with the foliar morphology monitored throughout the experimental period. The morphological changes and photos of the seedlings across the experiment are shown in **Table 2** and **Figure 2**, respectively.

**Table 2:** Temporal changes in the foliar and branch morphology of *Machilus thunbergii* seedlings under different nitrogen treatments.

Time/DAT	Sodium nitrate treatment	Urea treatment	Ammonium sulfate treatment	Control treatment
0–30	Normal growth	Normal growth	Normal growth	Normal growth
60	Leaf tips slightly scorched at 40 DAT; leaf tips and margins scorched and blackened at 60 DAT	Normal growth	Normal growth	Normal growth
90	Worst symptoms of scorching, no visible leaf drop	Slight scorching of leaf tips and margins	Leaf tips and margins show scorching symptoms to a greater extent	Normal growth
120	Almost total leaf scorch, with obvious defoliation	Increased leaf scorching symptoms	Increased leaf scorching symptoms	Normal growth
150	Seedlings with all leaves scorched on the whole plant and a lot of leaves withered off	Nearly a quarter of the seedlings showing symptoms of leaf scorching	Nearly 1/3 of the seedlings showing symptoms of leaf scorching	Normal growth

*DAT*, days after transplantation.

**Table 2** demonstrates that all nitrogen treatments induced varying degrees of fertilizer injury in *M. thunbergii* seedlings, with the symptom severity progressively intensifying over time. This phytotoxicity occurred because the initially elevated nitrogen concentration in the substrate was exacerbated by supplemental fertilization. Sodium nitrate-treated seedlings elicited the earliest and most severe symptoms, followed by the ammonium sulfate-treated seedlings. The urea-treated seedlings exhibited the mildest phytotoxic effects. No seedling mortality occurred by 150 DAT. Subsequent observation of the seedlings showed that the majority of them gradually sprouted new shoots in the basal stem portion.

### 3.2 Measurement results of *M. thunbergii* seedling growth indicators

#### 3.2.1 Determination of the height growth and the basal diameter growth of *M. thunbergii* seedlings in the different treatments

**Figure 3:**
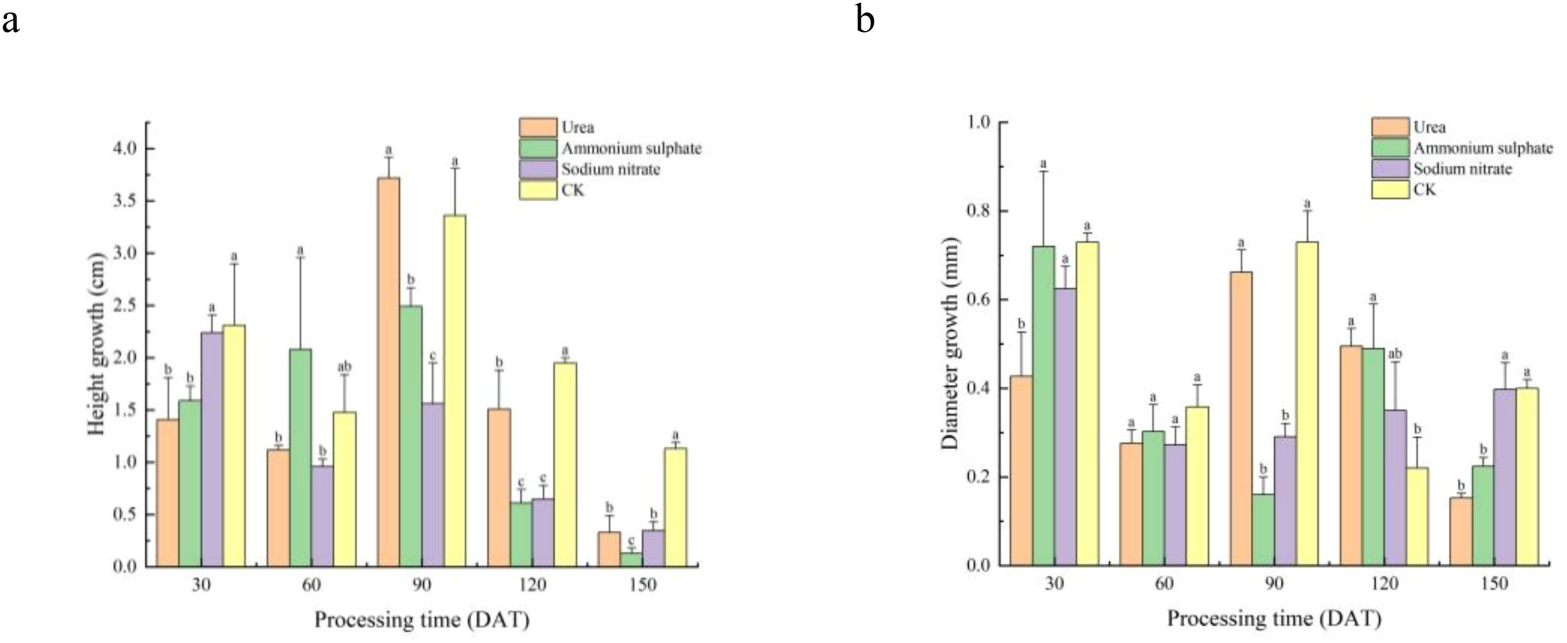
Temporal dynamics of *Machilus thunbergii* seedling growth under different nitrogen regimes. Increments in height growth **(A)** and diameter growth **(B)** across five measurement periods. For each time period, *different lowercase letters above bars* indicate significant differences among treatments (*p* < 0.05). Statistical comparisons are independent for each period.

The height and diameter increments of *M. thunbergii* seedlings are presented in **Table 3** and **Figure 3**. Throughout the experimental period, the performance of the different nitrogen fertilizer treatments exhibited significant stage specificity. In terms of height growth (**Figure 3A**), the CK group maintained a leading position across multiple stages (0–30 DAT and 90–150 DAT) and was significantly greater than all other fertilizer treatments during 90–150 DAT (0.01 < *p* < 0.05). However, the fertilizer treatments also demonstrated advantages during specific periods: the ammonium sulfate treatment achieved the maximum height increment at 30–60 DAT, while the urea treatment at 60–90 DAT was significantly higher than the other two nitrogen treatments (0.01 < *p* < 0.05), albeit not significantly different from the control (*p* > 0.05).

**Table 3:** Temporal patterns of the height and diameter growth of *Machilus thunbergii* seedlings across nitrogen treatments.

Treatment	30 DAT	60 DAT	90 DAT	120 DAT	150 DAT
Height growth (cm)					
Control	2.31 ± 0.59a	1.48 ± 0.36ab	3.36 ± 0.45a	1.95 ± 0.05a	1.13 ± 0.06a
Urea	1.41 ± 0.4b	1.12 ± 0.04b	3.72 ± 0.2a	1.51 ± 0.37b	0.33 ± 0.16b
Ammonium sulfate	1.59 ± 0.14b	2.08 ± 0.88a	2.49 ± 0.18b	0.61 ± 0.13c	0.13 ± 0.05c
Sodium nitrate	2.24 ± 0.17a	0.96 ± 0.07b	1.56 ± 0.39c	0.65 ± 0.13c	0.35 ± 0.08b
Diameter growth (mm)					
Control	0.73 ± 0.02a	0.36 ± 0.05a	0.73 ± 0.07a	0.22 ± 0.07b	0.40 ± 0.02a
Urea	0.43 ± 0.10b	0.28 ± 0.03a	0.66 ± 0.05a	0.50 ± 0.04a	0.15 ± 0.01b
Ammonium sulfate	0.72 ± 0.17a	0.30 ± 0.06a	0.16 ± 0.04b	0.49 ± 0.10a	0.22 ± 0.02b
Sodium nitrate	0.63 ± 0.05a	0.27 ± 0.04a	0.29 ± 0.03b	0.35 ± 0.11ab	0.40 ± 0.06a

For each time period, different lowercase letters indicate significant differences among treatments (*P* < 0.05). Statistical comparisons are independent for each period.

*DAT*, days after transplantation.

In terms of diameter growth, the control group yielded the greatest increment during 0–90 DAT. In the later growth stages, the effects of the nitrogen fertilizers became apparent: at 90–120 DAT, the diameter increment in the urea treatment was significantly greater than that in the control (0.01 < *p* < 0.05). At 120–150 DAT, the diameter increment in the sodium nitrate treatment peaked and was significantly higher than that in both the urea and ammonium sulfate treatments (0.01 < *p* < 0.05).

Under the fertilization level set in this experiment, the promoting effects of the nitrogen fertilizers on the growth of *M. thunbergii* seedlings were stage-specific and did not demonstrate a significant and sustained overall advantage over the control group.

#### 3.2.2 Determination of the biomass of *M. thunbergii* seedlings in the different treatments

The seedling biomass reflects the vegetative growth of seedlings and serves as a key quality indicator (Wei et al., 2018). The biomass measurements across treatments are shown in **Table 4**.

**Table 4:** Partitioning of the root, stem, and leaf biomass in response to nitrogen fertilizer treatments at the end of the experiment.

Treatment	Leaf biomass (g/plant)	Stem biomass (g/plant)	Root biomass (g/plant)	Whole-plant biomass (g/plant)
Control	24.99 ± 3.27Aa	16.96 ± 5.79Aa	13.86 ± 2.99Aa	55.81 ± 11.55Aa
Urea	24.02 ± 2.74Aa	20.18 ± 5.49Aa	16.15 ± 4.52Aa	60.35 ± 12.05Aa
Ammonium sulfate	16.09 ± 3.60Bb	16.51 ± 8.28Aa	15.70 ± 4.84Aa	48.30 ± 11.63Aa
Sodium nitrate	10.41 ± 5.85Bb	15.75 ± 5.67Aa	14.68 ± 3.61Aa	40.84 ± 3.52Aa

Values followed by different lowercase (*p* < 0.05) or uppercase (*p* < 0.01) letters within a column are significantly different.

As shown in **Table 4**, the urea treatment showed maximum values for the stem, root, and total biomass, increasing by 18.99%, 20.85%, and 8.13%, respectively, compared with the control. The seedlings in the sodium nitrate treatment had the lowest values across all biomass parameters. The stem and root biomass of the control seedlings accounted for 30.89% and 24.83% of the whole plant biomass, respectively, which were lower than those of the other three nitrogen treatments. All three nitrogen treatments enhanced the stem and root biomass (which was most pronounced with urea); however, they reduced the leaf biomass.

### 3.3 Determination of the root indexes of *M. thunbergii* seedlings in the different treatments

The results of the seedling root index measurements for each treatment are shown in **Table 5**.

**Table 5:** Root system morphological traits of *Machilus thunbergii* seedlings under different nitrogen treatments.

Treatment	Total root length (cm)	Root surface area (cm^2^)	Root volume (cm^3^)	Mean root basal diameter (mm)	Total no. of root tips
Control	2,278.85 ± 439.78Aa	899.31 ± 195.97Ab	64.48 ± 19.70Bb	1.58 ± 0.23Aab	738 ± 63Bb
Urea	2,690.30 ± 541.07Aa	1,293.74 ± 212.11Aa	132.60 ± 24.66Aa	1.99 ± 0.44Aa	1,228 ± 223Aa
Ammonium sulfate	2,288.24 ± 86.16Aa	966.80 ± 38.56Ab	67.10 ± 7.57Bb	1.42 ± 0.08Ab	933 ± 128ABb
Sodium nitrate	2,097.39 ± 225.80Aa	938.18 ± 108.93Ab	70.00 ± 7.96Bb	1.54 ± 0.13Aab	906 ± 84ABb

Values followed by different lowercase (*p* < 0.05) or uppercase (*p* < 0.01) letters within a column are significantly different.

As shown in **Table 5**, the urea-treated seedlings showed maximal root system values: the total root length (2,690.30 cm) exceeded those in the ammonium sulfate and sodium nitrate treatments by 17.57% and 28.27%, respectively, representing 1.18-fold of the control. The root surface area, the root volume, and the total number of root tips of the seedlings in this nitrogen treatment were significantly higher than those in the other nitrogen treatments (0.01 < *p* < 0.05), which were 1,293.74 cm^2^, 132.60 cm^3^, and 1,228, respectively, and showing 43.86%, 105.65%, and 66.40% increases over the control, respectively. The mean root basal diameter of the urea-treated seedlings was 1.99 mm, showing a 40.14% increase over the ammonium sulfate-treated seedlings, with the difference reaching a significant level (*p <* 0.05). The above analyses showed that urea could effectively promote the root growth of *M. thunbergii* seedlings at this level of nitrogen application, while the ammonium sulfate and sodium nitrate treatments showed negligible effects.

### 3.4 Determination of the nitrogen metabolism enzyme activity in the leaves of *M. thunbergii* seedlings with the different treatments

#### 3.4.1 Determination of the leaf NR activity of *M. thunbergii* seedlings with the different treatments

**Figure 4:**
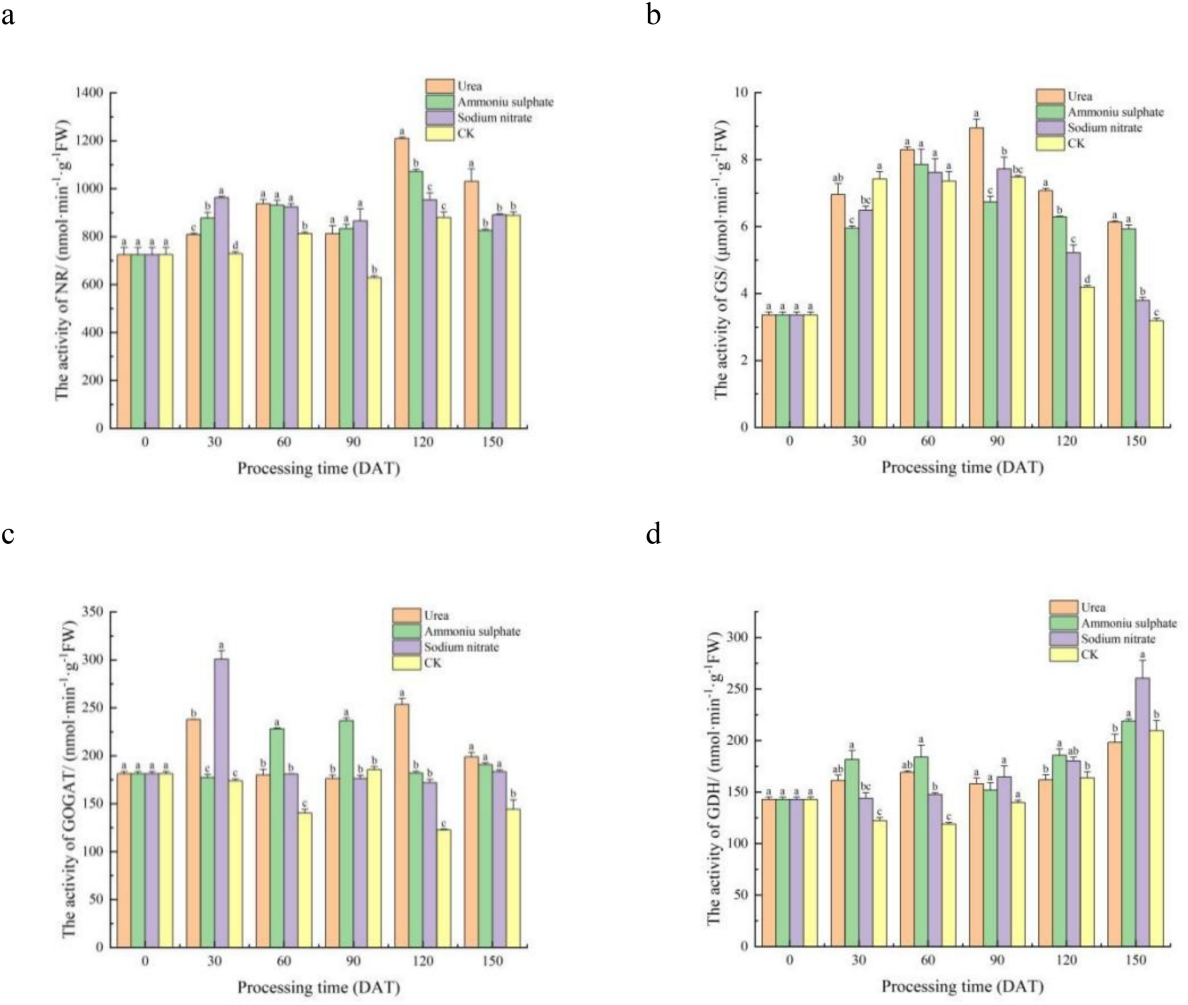
Time course changes in the leaf nitrogen metabolism enzyme activities of *Machilus thunbergii* seedlings under different nitrogen fertilizers. Nitrate reductase (NR) **(A)**, glutamine synthetase (GS) **(B)**, glutamate synthase (GOGAT) **(C)**, and glutamate dehydrogenase (GDH) **(D)** were measured at five time points. *Different lowercase letters within a time point* indicate significant differences among treatments (*p* < 0.05).

The results of the leaf NR activity in the seedlings of each treatment are shown in **Table 6**. **Figure 4A** reveals that, from 30 to 90 DAT post-treatment, the leaf NR activity in each nitrogen fertilizer treatment was significantly higher than that of the control seedlings (*p <* 0.05), indicating that the nitrogen fertilizer had a significant promoting effect on the NR activity at this application level during this period. At 120 DAT, the seedlings treated with urea and ammonium sulfate had significantly higher leaf NR activity than those in the sodium nitrate and control treatments (0.01 < *p* < 0.05). By 150 DAT, the urea treatment maintained significantly superior NR activity compared with all the other groups (0.01 < *p* < 0.05). Throughout the experiment, all treatments exhibited bimodal fluctuations in the leaf NR activity over time. The first peak of NR activity in the seedlings in the sodium nitrate treatment appeared at 30 DAT, while those in the urea and ammonium sulfate treatments appeared at 60 DAT. The second peak of NR activity in each nitrogen application treatment appeared at 120 DAT. These results indicate that the leaf NR activity of *M. thunbergii* seedlings is co-regulated by the nitrogen source and the treatment duration. The promoting effect was phase-specific, with urea demonstrating a more pronounced and sustained induction of NR activity, particularly at the later stage of the experiment.

**Table 6:** Temporal dynamics of the activities of the key nitrogen metabolism enzymes in the leaves of *Machilus thunbergii* seedlings across nitrogen treatments.

Nitrogen Metabolism Enzymes	Treatment	0 DAT	30 DAT	60 DAT	90 DAT	120 DAT	150 DAT
NR activity (nmol min^−1^ g^−1^ FW)	Control	724.91 ± 41.57a	729.01 ± 12.15d	812.32 ± 9.86b	629.01 ± 11.63b	880.3 ± 32.48c	888.92 ± 19.56b
	Urea	724.91 ± 41.57a	809.11 ± 6.67c	937.42 ± 25.62a	812.61 ± 47.58a	1209.54 ± 7.80a	1030.18 ± 74.05a
	Ammonium sulfate	724.91 ± 41.58a	877.43 ± 33.83b	931.26 ± 28.02a	833.8 ± 25.43a	1071.44 ± 13.89b	825.96 ± 7.65b
	Sodium nitrate	724.91 ± 41.59a	962.94 ± 7.20a	924.02 ± 19.36a	866 ± 70.47a	953.91 ± 41.52c	891.42 ± 6.15b
GS activity (μmol min^−1^ g^−1^ FW)	Control	3.36 ± 0.13a	7.42 ± 0.31a	7.36 ± 0.39a	7.48 ± 0.06bc	4.19 ± 0.07d	3.19 ± 0.09c
	Urea	3.36 ± 0.13a	6.96 ± 0.45ab	8.3 ± 0.09a	8.95 ± 0.37a	7.07 ± 0.10a	6.13 ± 0.05a
	Ammonium sulfate	3.36 ± 0.13a	5.95 ± 0.09c	7.86 ± 0.64a	6.74 ± 0.24c	6.29 ± 0.03b	5.93 ± 0.17a
	Sodium nitrate	3.36 ± 0.13a	6.49 ± 0.18bc	7.62 ± 0.58a	7.72 ± 0.50b	5.22 ± 0.32c	3.79 ± 0.13b
GOGAT activity (nmol min^−1^ g^−1^ FW)	Control	181.24 ± 3.62a	173.94 ± 2.84c	140.19 ± 5.73c	185.61 ± 4.11b	122.61 ± 1.09c	144.25 ± 13.88b
	Urea	181.24 ± 3.62a	237.8 ± 0.29b	180.05 ± 8.27b	176.19 ± 5.12b	253.47 ± 8.86a	198.71 ± 6.85a
	Ammonium sulfate	181.24 ± 3.62a	177.13 ± 4.8c	228.18 ± 0.90a	236.65 ± 4.08a	181.89 ± 2.97b	190.59 ± 3.18a
	Sodium nitrate	181.24 ± 3.62a	300.84 ± 12.44a	180.97 ± 0.27b	176.22 ± 4.68b	171.79 ± 5.30b	183.31 ± 3.01a
GDH activity (nmol min^−1^ g^−1^ FW)	Control	142.95 ± 2.91a	122.14 ± 4.44c	119.06 ± 2.06c	139.69 ± 3.53a	163.7 ± 8.45b	209.65 ± 13.79b
	Urea	142.95 ± 2.91a	161.31 ± 7.13ab	169.21 ± 2.00ab	158.06 ± 7.87a	161.81 ± 6.65b	198.13 ± 11.16b
	Ammonium sulfate	142.95 ± 2.91a	181.63 ± 11.49a	184.08 ± 16.27a	151.85 ± 9.87a	185.72 ± 8.80a	218.9 ± 2.87ab
	Sodium nitrate	142.95 ± 2.91a	143.99 ± 7.8bc	147.72 ± 2.19b	164.77 ± 15.14a	180.35 ± 5.01ab	260.56 ± 24.45a

Different lowercase letters within a time point indicate significant differences among treatments (*p* < 0.05).

*DAT*, days after transplantation; *FW*, fresh weight; *NR*, nitrate reductase; *GS*, glutamine synthetase; *GOGAT*, glutamate synthetase; *GDH*, glutamate dehydrogenase.

#### 3.4.2 Determination of the leaf GS activity of *M. thunbergii* seedlings with the different treatments

The results of the leaf GS activity in the seedlings of each treatment are shown in **Table 6**. **Figure 4B** shows that, at 30 DAT, the leaf GS activity of the seedlings in each nitrogen fertilizer treatment was observably lower than that of the control, although the difference was not statistically significant (*p* > 0.05). From 30 to 120 DAT, all treatments displayed a unimodal pattern in leaf GS activity, initially increasing and then decreasing. The leaf GS activity of the seedlings treated with ammonium sulfate reached its peak at 60 DAT, while those of the seedlings treated with urea and sodium nitrate reached their peaks at 90 DAT. At 150 DAT, the leaf GS activity of the seedlings in the three nitrogen treatments was significantly higher than that of the control (*p* < 0.05). These results demonstrate that the leaf GS activity in *M. thunbergii* seedlings was co-regulated by the nitrogen fertilizer type and the application duration. Specifically, while nitrogen fertilization initially suppressed the GS activity relative to the control, it ultimately induced a significant and sustained enhancement by the end of the experiment, with different nitrogen sources triggering distinct temporal patterns in peak activity.

#### 3.4.3 Determination of the leaf GOGAT activity of *M. thunbergii* seedlings with the different treatments

The results of the leaf GOGAT activity in the seedlings of each treatment are shown in **Table 6**. **Figure 4C** illustrates distinct temporal dynamics in the GOGAT activity among the nitrogen treatments. Specifically, the activity in the ammonium sulfate- and sodium nitrate-treated seedlings followed a unimodal pattern, whereas a bimodal pattern was observed in the urea-treated seedlings. At 30 DAT, the leaf GOGAT activity in the seedlings of both the urea and sodium nitrate treatments reached the first peak and was significantly higher than that of the control (0.01 < *p* < 0.05). No significant difference was observed between the ammonium sulfate treatment and the control (*p* > 0.05). By 60 DAT, the ammonium sulfate treatment exhibited significantly higher GOGAT activity than all the other treatments (0.01 < *p* < 0.05), and this superiority persisted at its peak (90 DAT). A second pronounced peak occurred at 120 DAT in the urea-treated seedlings, which was significantly higher than all other nitrogen treatments (0.01 < *p* < 0.05). Throughout the experiment, the leaf GOGAT activity of the seedlings in the three nitrogen treatments showed higher values than that of the control. This result indicates that the changes in the GOGAT activity in *M. thunbergii* seedling leaves are co-regulated by the nitrogen fertilizer type and the application duration, exhibiting a complex temporal pattern. Among the fertilizers tested, urea was the most effective in enhancing the activity of GOGAT, inducing two significant peaks over the experimental period.

#### 3.4.4 Determination of the leaf GDH activity of *M. thunbergii* seedlings with the different treatments

The results of the leaf GDH activity of the seedlings in each treatment are shown in **Table 6**. Statistical analysis of the temporal pattern (**Figure 4D**) revealed that the GDH activity was relatively stable during the early stage (0–60 DAT), with no consistent significant differences observed among the treatments. The ranking of GDH activity among treatments varied across time points: at 30 and 60 DAT, the order was ammonium sulfate > urea > sodium nitrate > the control, while by 90 DAT, it shifted to sodium nitrate > urea > ammonium sulfate > the control. At 120 DAT, the ammonium sulfate treatment reached its peak GDH activity, which was significantly higher than that of the control (0.01 < *p* < 0.05). By the end of the experiment (150 DAT), all nitrogen treatments had reached their peak leaf GDH activity. Seedlings with the sodium nitrate treatment showed the highest leaf GDH activity, differing significantly from those in the control and urea treatments (0.01 < *p* < 0.05). Compared with that in the control, the leaf GDH activity in the ammonium sulfate, sodium nitrate, and urea treatments was increased by 4.41%, 24.28%, and 5.49%, respectively.

This analysis demonstrates that the leaf GDH activity in *M. thunbergii* seedlings was influenced by both the nitrogen fertilizer type and the treatment duration, with the effect of fertilizer type being more pronounced in the early stages and the effect of duration becoming dominant later. Overall, all three nitrogen sources enhanced the GDH activity by the end of the experiment, with sodium nitrate showing the greatest relative increase.

### 3.5 Accumulation and transport of nitrogen by *M. thunbergii* seedlings under the different nitrogen fertilizer treatments

#### 3.5.1 Determination of the nitrogen content and accumulation in different parts of *M. thunbergii* seedlings

**Figure 5:**
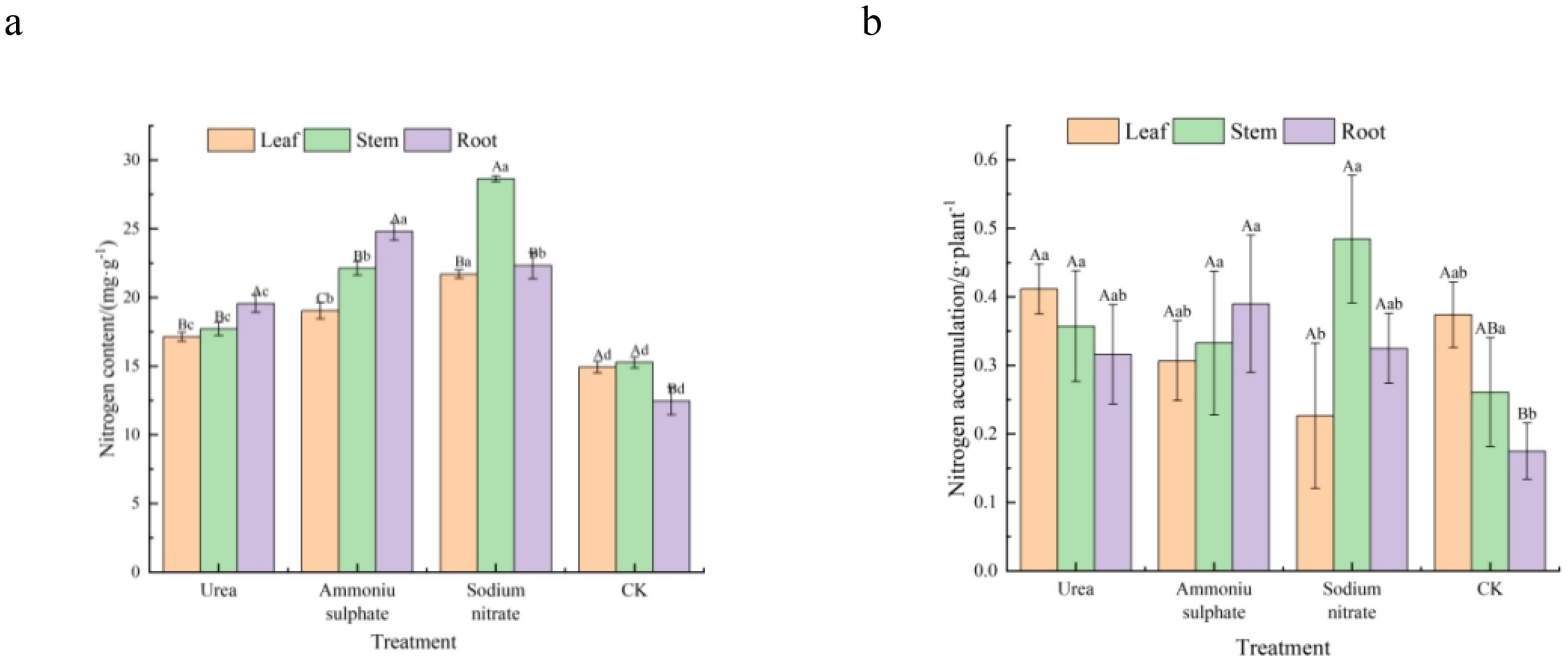
Organ-specific nitrogen allocation in *Machilus thunbergii* seedlings as affected by nitrogen fertilization at the final harvest. Nitrogen content **(A)** and nitrogen accumulation **(B)** in the roots, stems, and leaves. *Different lowercase letters above bars* for a given plant part indicate significant differences among treatments (*p* < 0.05).

**Table 7** and **Figure 5** present the results of the nitrogen content and accumulation (calculated with Equation 1) in the roots, stems, and leaves of *M. thunbergii* seedlings across treatments. As shown in **Figure 5A**, there were significant differences (*p* < 0.05) between the nitrogen contents in the leaf, stem, and root parts of *M. thunbergii* seedlings for the different nitrogen fertilizer treatments, and the total nitrogen content in the seedlings of each nitrogen treatment followed the order: sodium nitrate treatment > ammonium sulfate treatment > urea treatment > the control. Compared with the seedlings in the control group, those treated with urea, ammonium sulfate, and sodium nitrate increased their nitrogen content by 14.66%–56.86% in the leaves, 27.44%–99.04% in the stems, and 45.25%–87.43% in the roots, respectively. For nitrogen accumulation in each part of the seedlings, the leaf part of the urea-treated seedlings showed maximum accumulation, while that of the sodium nitrate-treated seedlings had the minimum. No significant difference (*p* > 0.05) was observed in the stem nitrogen accumulation among treatments (*p* > 0.05). The nitrogen accumulation in the root part of the seedlings in each nitrogen treatment was higher than that of the control, with the nitrogen accumulation in the root part of the seedlings treated with ammonium sulfate exhibiting the maximum value. In both the urea and control treatments, nitrogen accumulation exhibited the following pattern: leaf > stem > root. However, the ammonium sulfate and sodium nitrate treatments showed peak nitrogen accumulation in the roots and stems, respectively, which was due to the phytotoxicity from the two treatments: a large number of leaves fell off. These results indicate that the application of nitrogen fertilizers increases the nitrogen content in all parts of *M. thunbergii* seedlings, but that ammonium sulfate and sodium nitrate treatments adversely affect the leaf nitrogen accumulation.

**Table 7:** Nitrogen content and accumulation in the different organs of *Machilus thunbergii* seedlings at the final harvest.

Organ Treatment	Nitrogen content (mg g^−1^)			Nitrogen accumulation (g/plant)		
	Leaf	Stem	Root	Leaf	Stem	Root
Control	14.94 ± 0.52Ad	15.28 ± 0.48Ad	12.47 ± 1.25Bd	0.37 ± 0.06Aab	0.26 ± 0.10ABa	0.17 ± 0.05Bb
Urea	17.13 ± 0.40Bc	17.71 ± 0.57Bc	19.56 ± 0.79Ac	0.41 ± 0.04Aa	0.36 ± 0.10Aa	0.32 ± 0.09Aab
Ammonium sulfate	19.04 ± 0.74Cb	22.13 ± 0.63Bb	24.82 ± 0.79Aa	0.31 ± 0.07Aab	0.37 ± 0.19Aa	0.39 ± 0.12Aa
Sodium nitrate	21.7 ± 0.37Ba	28.64 ± 0.26Aa	22.33 ± 1.19Bb	0.23 ± 0.13Ab	0.45 ± 0.16Aa	0.32 ± 0.06Aab

Values followed by different lowercase (*p* < 0.05) or uppercase (*p* < 0.01) letters within a column are significantly different.

#### 3.5.2 Determination of the nitrogen translocation capacity of *M. thunbergii* seedlings

**Figure 6:**
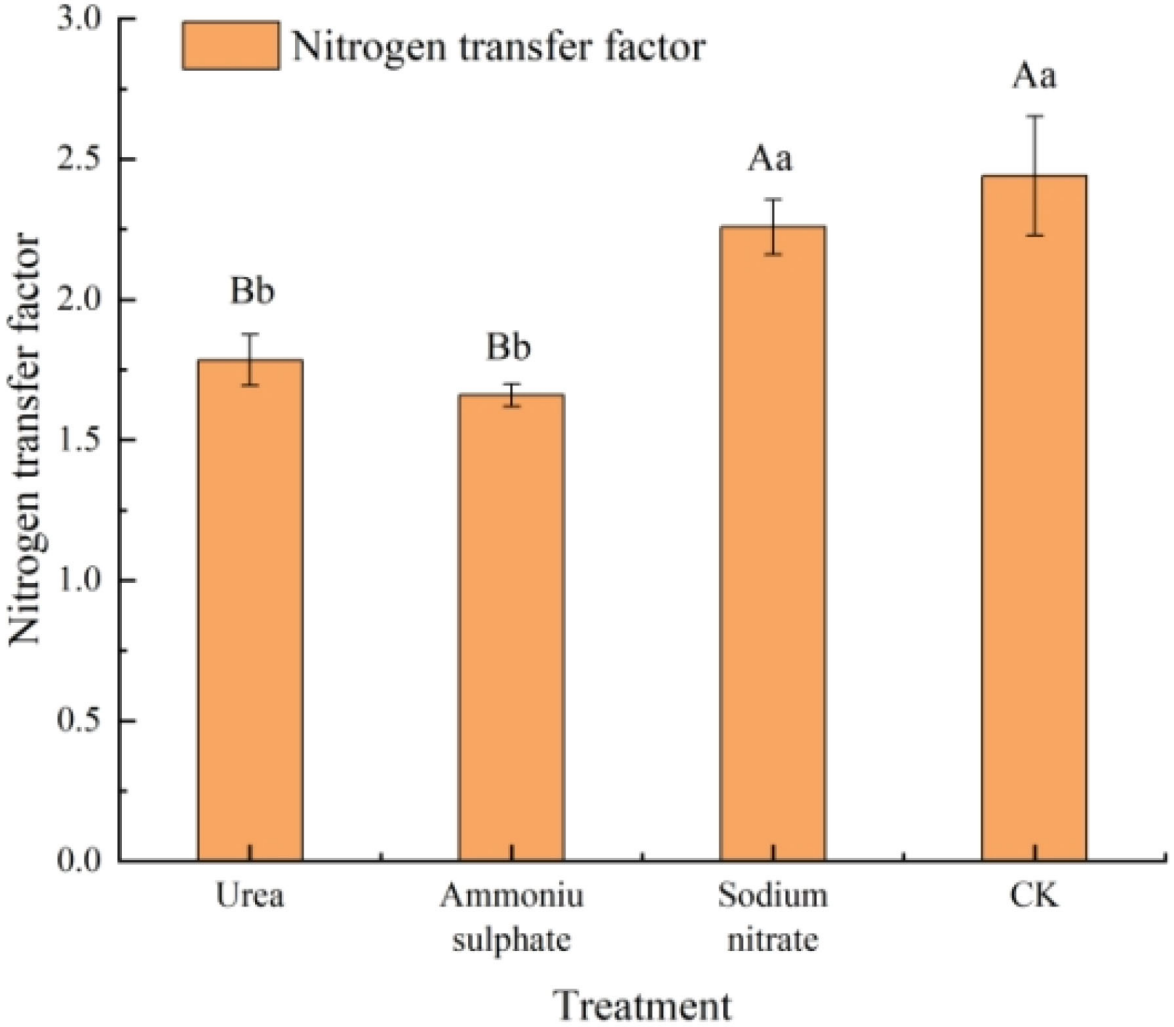
Nitrogen transfer factor of *Machilus thunbergii* seedlings in response to nitrogen fertilizer treatments. The transfer factor was calculated as the aboveground-to-root nitrogen content ratio. *Different lowercase letters indicate* significant differences among treatments (*p* < 0.05).

The nitrogen transport capacity of the seedlings in each nitrogen treatment was calculated with Equation 2, and the results are shown in **Figure 6** and **Table 8**. All nitrogen treatments exhibited lower nitrogen transfer factor compared with the control. The sodium nitrate treatment showed no significant difference from the control (*p* > 0.05), while the urea and ammonium sulfate treatments showed significantly lower values than the control (*p* < 0.01). The ammonium sulfate treatment yielded the minimum nitrogen transfer factor of 1.66. These results indicate that the nitrogen treatments do not enhance the nitrogen transport from the roots to the shoots; however, they improve the root nitrogen uptake, with ammonium sulfate showing the strongest promoting effect.

**Table 8:** Nitrogen transfer factor of *Machilus thunbergii* seedlings in response to nitrogen fertilizer treatments.

Treatment	Transfer factor
Control	2.44 ± 0.26Aa
Urea	1.78 ± 0.11Bb
Ammonium sulfate	1.66 ± 0.05Bb
Sodium nitrate	2.26 ± 0.12Aa

Values followed by different lowercase (*p* < 0.05) or uppercase (*p* < 0.01) letters within a column are significantly different.

### 3.6 Correlation analysis of the indexes of *M. thunbergii seedlings* in the different treatments

**Figure 7:**
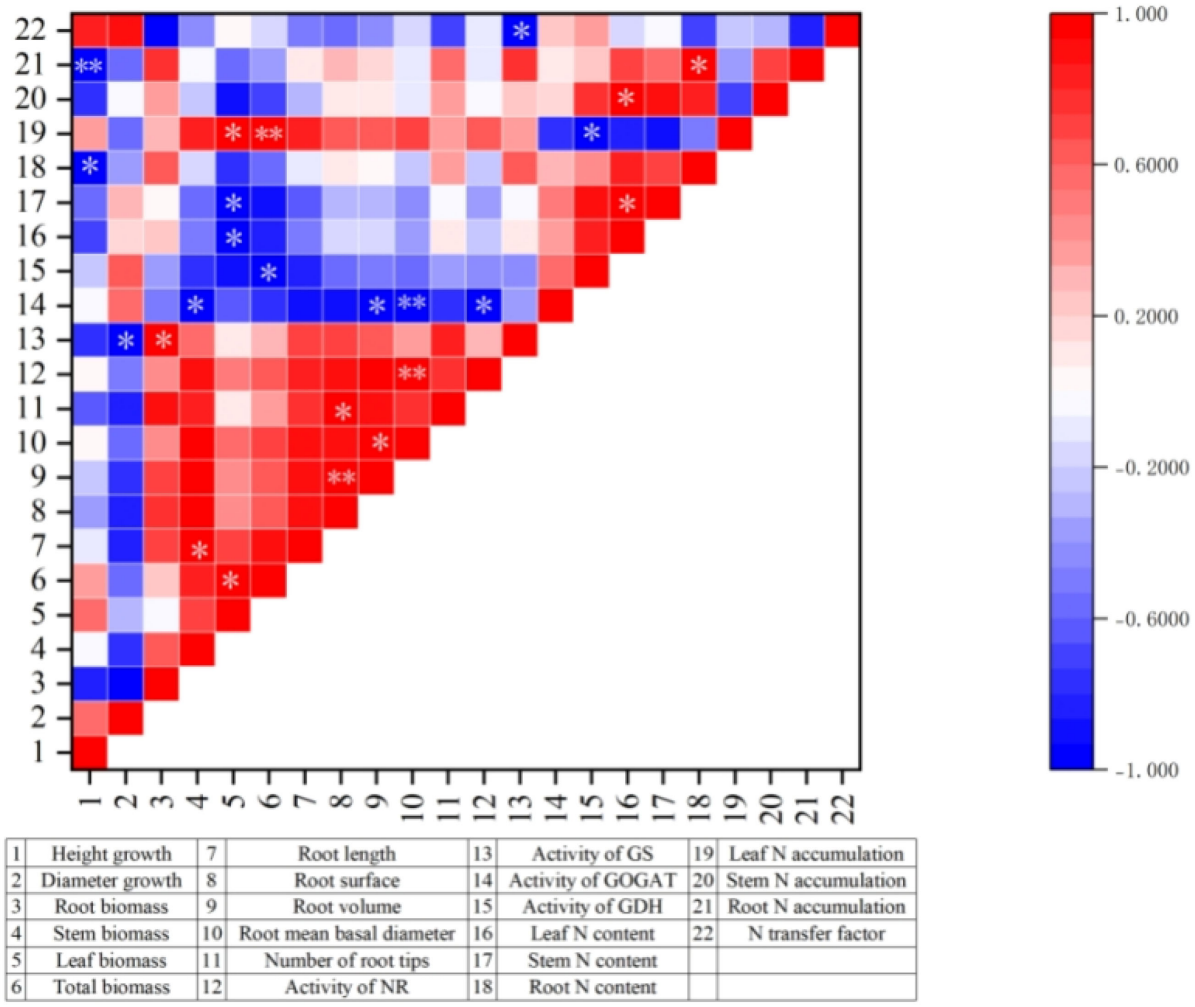
Correlations among final trait values in *Machilus thunbergii* seedlings across fertilization treatments. *Asterisk* indicates that the correlation is significant at the 0.05 level (two-tailed). *Two asterisks* represent significance at the 0.01 level (two-tailed).

To elucidate the macro-level associative patterns among traits, a correlation analysis was performed based on the treatment means of the various parameters for *M. thunbergii* seedlings under the different fertilization treatments. As shown in **Figure 7**, positive correlations were predominantly observed among several key clusters: between the biomass and root system parameters, in mutual correlations between the root parameters, between the biomass and enzyme activities, and between the nitrogen content and nitrogen accumulation. These interrelated associations collectively underpin the phenotypic expression of robust shoot and root growth, reflecting an integrated physiological foundation of a highly efficient nitrogen metabolism and a strategic resource allocation (Shipley and Meziane, 2002; Fan et al., 2025). The synergistic coordination between organ growth and nitrogen metabolism function is comprehensively embodied in these positive correlations.

In contrast, negative correlations were primarily concentrated between the growth indicators and the nitrogen metabolism components, including the key enzyme and nitrogen content/accumulation in various tissues. This suggests that, during periods of rapid growth, *M. thunbergii* seedlings tend to preferentially allocate assimilated resources to structural growth rather than to storage functions, reflecting a strategic resource allocation trade-off (Li et al., 2006).

Overall, the correlation analysis revealed tightly coupled synergistic and trade-off relationships among nutrient allocation, root system architecture, and the nitrogen metabolism system in response to the different nitrogen fertilizers, presenting a coherent physiological response profile. Consequently, the seedling response is a systematic process, representing the integrated outcome of an internal resource allocation and a balance between organ growth and nitrogen metabolic function.

## 4 Discussion

### 4.1 Effects of different nitrogen fertilizers on the growth and root morphology of *M. thunbergii* seedlings

Nitrogen fertilizer application has a significant impact on plant growth with species-specific requirements. Appropriate nitrogen application that meets plant needs promotes growth, whereas improper application restricts development (Su et al., 2010). Single nitrogen application significantly promotes plant growth at the early stage; however, this exhibits an inhibitory effect on plant growth with prolonged treatment (Sun et al., 2018). In this experiment, all three nitrogen fertilizers applied at 3 g per plant induced alterations in the leaves and roots of *M. thunbergii* seedlings, with the most severe symptoms observed under sodium nitrate treatment.Height and diameter growth are reliable indicators of the seedling growth status (Zhu et al., 2018). Studies on *Cinnamomum camphora* have demonstrated that an appropriate nitrogen application rate promotes growth in the seedling height and diameter (Dong E.Y, et al., 2025). Similarly, research on *Machilus pingii* reported a pattern of initial increase followed by a decrease in these growth metrics with increasing application of nitrogen (Deng et al., 2020). In contrast, the present study found that the height and diameter growth of *M. thunbergii* seedlings under the three different nitrogen fertilizer treatments were consistently lower than those in the control group. This indicates that the applied nitrogen concentrations are supra-optimal, leading to growth inhibition. The collective findings from these studies delineated a spectrum of growth responses to nitrogen fertilization. Furthermore, this experiment provided a basis for the selection of the most suitable fertilizer type for *M. thunbergii* under high-nitrogen stress conditions. The results of this experiment are in line with studies in *Hovenia acerba* (Zhang et al., 2018), *Atropa belladonna* (Wei et al., 2017), and *Nicotiana tabacum* (Xing and Ma, 2016).

The accumulation of biomass enhances plant tissue nutrient storage, demonstrating the influence of external conditions on plant growth (Yang et al., 2020). Research on *C. camphora* demonstrated that appropriate nitrogen application can enhance the biomass accumulation across plant parts and increase the proportional biomass allocation to stems and leaves (Dong et al., 2025). The study on *M. pingii* further revealed that the biomass in all components exhibited an initial increasing and then decreasing trend with increased nitrogen application (Deng et al., 2020). In the present experiment, however, none of the three nitrogen fertilizers significantly increased the total biomass accumulation of *M. thunbergii* seedlings compared with the control (*p* > 0.05). This indicates that the dosage of 3 g nitrogen per seedling is ineffective in altering biomass accumulation. Collectively, these findings outline a continuum of nitrogen response patterns: from a significant promotion in *C. camphora*, to a hump-shaped response in *M. pingii*, and finally to no significant response in *M. thunbergii* under the given conditions.

Notably, urea treatment resulted in increased biomass compared with the other nitrogen forms, which is likely linked to an optimized carbon–nitrogen balance. Yang et al. (2008) postulated that nitrogen fertilization enhances plant biomass primarily by boosting the photosynthetic capacity. The authors suggested that nitrogen application triggers a response mechanism in mesophyll cells, promoting chlorophyll synthesis and increasing its content, which in turn facilitates photosynthesis. In this experiment, the increased biomass of the *M. thunbergii* seedlings under urea treatment is likely linked to an optimized carbon–nitrogen balance. Urea presumably supplied ample nitrogenous compounds, such as chlorophyll and key photosynthetic enzymes, thereby directly enhancing the leaf photosynthetic capacity. The consequent increase in photosynthetic products (carbon skeletons) could then provide sustained energy and substrates for nitrogen uptake and assimilation (Yan et al., 2025). This positive feedback loop between carbon and nitrogen metabolism may represent the fundamental physiological basis for the significant biomass accumulation observed in the urea-treated seedlings.

In terms of root development, all three nitrogen treatments exhibited superior root morphology compared with the control, indicating that all three nitrogen fertilizers significantly enhanced root development, with the following efficacy ranking: urea treatment > ammonium sulfate treatment > sodium nitrate treatment. This is consistent with the findings for *Paeonia lactiflora* (Wang et al., 2025). In this experiment, the sodium nitrate treatment resulted in lower values for many of the seedling growth indicators compared with the control group. However, the same treatment led to greater values for root surface area, root volume, and total root tip number compared with the control, indicating a dichotomous effect: at the application rate of 3 g per seedling, sodium nitrate appears to exert phytotoxic effects on the overall growth, while its nitrate nitrogen component specifically promotes root system development. This phenomenon is consistent with the research by Zhang et al. (2024) on *Ormosia henryi* seedlings. Furthermore, the seedlings treated with urea exhibited superior root morphology compared with those treated with ammonium sulfate. This suggests that this fertilization level of amide nitrogen fertilizers enhances root development more effectively than ammonium nitrogen fertilizers. A similar pattern of differential growth responses to nitrogen forms was reported by Zhang et al. (2023) on *Gossypium hirsutum*. The final correlation analysis elucidated why the urea-treated seedlings exhibited superior height, diameter, and biomass compared with the other treatments. The significant positive correlation between biomass and the root system parameters indicates that the vigorous growth of the seedlings results from a positive feedback loop involving a highly efficient nitrogen assimilation and a well-developed root system.

### 4.2 Effects of the different nitrogen fertilizer treatments on the activity of nitrogen-metabolizing enzymes in the leaves of *M. thunbergii* seedlings

Numerous studies have shown that nitrogen fertilization affects the activity of the nitrogen metabolic enzymes in plants (Ning et al., 2019). Zhao et al. (2017) demonstrated that moderate nitrogen fertilization enhances the leaf NR and GS activities within optimal ranges; however, these activities decline when nitrogen exceeds threshold levels. This is reflected in this study. For instance, the leaf NR activity in the nitrogen-treated seedlings was generally higher than that in the control from 0 to 120 DAT; however, by 150 DAT, the activity in the ammonium sulfate treatment had fallen below that of the control. As for the leaf GS activity, all of the nitrogen treatments surpassed the control at 60 DAT, but with the ammonium sulfate treatment dropping below the control by 90 DAT, with a general declining trend in the GS activity observed over time across all nitrogen treatments. The results of this study indicate that the sodium nitrate treatment generally results in a higher NR activity compared with the ammonium sulfate treatment, suggesting that, at 3 g per plant, nitrate nitrogen has a better effect on the leaf NR activity than ammonium nitrogen. The experiment conducted by Zhang et al. (2024) yielded the same results. GS and GOGAT are the key enzymes in the plant nitrogen assimilation pathway, the “GS/GOGAT pathway,” and their activities directly reflect the nitrogen assimilation efficiency (Liu et al., 2011). Compared with other nitrogen fertilizers, an appropriate increase in ammonium nitrogen application can promote the plant GS activity at the same nitrogen level, in turn enhancing nitrogen metabolism (Yang et al., 2007). In the later stage of the experiment, the GS and GOGAT activities in the seedling leaves in the ammonium sulfate treatment were higher than that in the sodium nitrate treatment, indicating that, compared with nitrate nitrogen, ammonium nitrogen is beneficial for improving the leaf GOGAT activity. Zhang et al. (2017) believed that increasing the ratio of NH_4_^+^–N/NO_3_^−^–N in fertilizers can effectively enhance the GOGAT activity in seedling leaves. These two results mutually confirm each other. Throughout the experimental period, the urea-treated *M. thunbergii* seedlings exhibited the highest leaf GS enzyme activity, alongside superior growth performance (i.e., seedling height, ground diameter, and biomass) and the most developed root system architecture among the three nitrogen fertilizer treatments. This correlation suggests a synergistic relationship among these parameters. A superior root system architecture can significantly enhance the nitrogen uptake capacity of seedlings (Ren et al., 2021; Jing et al., 2023). To accommodate the increased nitrogen influx, the seedlings likely upregulated key assimilatory enzymes such as GS for efficient nitrogen assimilation (Tang et al., 2023). This process ultimately provided ample foundational materials for rapid plant growth, resulting in a significant growth advantage. It can be inferred that the promoting effect of urea treatment on *M. thunbergii* seedlings is systematic. Urea application enhances root development, and the resulting improved root system architecture not only ensures a stable nitrogen uptake but also supports the efficient and stable operation of the enzymatic systems for nitrogen metabolism in the leaves, thereby optimizing the overall growth performance of the plant. This systemic promoting mechanism is supported by the results of the correlation analysis in this study. The data revealed significantly positive correlations between the root biomass and GS activity, as well as between the root mean basal diameter and NR activity, indicating that modifications in the root morphology are closely linked to nutrient absorption and the overall nitrogen metabolism of the seedlings. Both the root mean diameter and the root volume showed significant negative correlations with the GOGAT activity, suggesting that specific changes in the root architecture may involve complex trade-offs with nitrogen assimilation. This aspect warrants further in-depth investigation.

### 4.3 Effects of the different nitrogen fertilizers on the nitrogen accumulation and translocation in *M. thunbergii* seedlings

The nitrogen absorption and translocation in plants can be characterized by indicators such as nitrogen content and nitrogen accumulation (Lian et al., 2022). Studies have shown that the application of nitrogen fertilizer has a significant impact on the nitrogen accumulation of plants, and increasing the application of nitrogen fertilizer can enhance the nitrogen accumulation in the aboveground part (Meng et al., 2025). In this study, nitrogen fertilizer application significantly increased both the total nitrogen content and the nitrogen accumulation in all tissues of *M. thunbergii* seedlings compared with the control. This indicates that all three nitrogen fertilizers effectively enhance the nitrogen uptake and accumulation at the 3-g per plant dosage, a finding consistent with previous research. The study by Han et al. (2025) on *Avena sativa* found that the nitrogen accumulation of plants with nitrogen applied at different concentrations all exceeded the control, which is similar to that in our study. The leaf nitrogen accumulation of the urea-treated seedlings was higher than that of the control, but that of the seedlings treated with ammonium sulfate and sodium nitrate was lower than that of the control. This could be due to the late stage of the experiment. Compared with the urea treatment, the ammonium sulfate and sodium nitrate treatments induced a more severe toxicity, leading to leaf wilting and a marked reduction in leaf biomass. This indicates that the application of urea is more effective for the uptake and accumulation of nitrogen in *M. thunbergii* seedlings at the 3-g per seedling application rate. The study by Dang et al. (2013) on *Triticum aestivum* found that, during the growth period, the nitrogen accumulation in the leaves of the plant was greater than that in the stems, which is consistent with the results of the experiment on *M. thunbergii*. These comparative findings are summarized in the table below. In this study, the nitrogen transfer factor of the *M. thunbergii* seedlings in the nitrogen treatments was all lower than that of the control, indicating that, at the 3-g per seedling application rate, the three nitrogen fertilizer types do not enhance the nitrogen transfer capacity of the seedlings, but improve their root nitrogen uptake. The seedlings treated with ammonium sulfate had the lowest transfer factor among the three nitrogen fertilizer treatments, indicating that, at this fertilization level, ammonium nitrogen has the greatest inhibitory effect on nitrogen transport in seedlings. This inhibitory effect represents one aspect of the regulatory role of nitrogen metabolism in plant growth. The correlation analysis in this study further elucidated the dual nature of this regulation. On the one hand, the positive correlation between GS activity and biomass indicates a driving effect of the nitrogen assimilation capacity on seedling growth. On the other hand, the significant negative correlation between height growth and root nitrogen content reveals a trade-off in resource allocation between supporting seedling growth and maintaining the nitrogen reserves. It is precisely this negative correlation pattern that explains why the height and the diameter growth of the seedlings in the three nitrogen treatment groups were lower than those in the control group, thereby providing insights into the resource allocation strategy of *M. thunbergii* in nitrogen-enriched environments.

## 5 Conclusion

At the 3-g per seedling application rate, all three nitrogen fertilizers produced a spectrum of toxicity in *M. thunbergii* seedlings. The effects of the different nitrogen fertilizer treatments on *M. thunbergii* seedlings were compared and summarized in **Table 9**. Although the application of nitrogen fertilizers inhibited the growth in the height and basal diameter of the seedlings, even triggering leaf scorching (with sodium nitrate being the most detrimental and urea the least), they conversely promoted root development. Consequently, the root morphology and biomass in all treatment groups surpassed those of the control. The seedlings treated with urea showed an increase in biomass, while those treated with ammonium sulfate and sodium nitrate did not exhibit this phenomenon. Therefore, sodium nitrate had the greatest toxic effect on *M. thunbergii* seedlings, followed by ammonium sulfate and urea. Among the nitrogen fertilizers tested, urea proved superior at the application rate of 3 g per plant for 3-year-old *M. thunbergii* seedlings. Nitrogen fertilization had dose-dependent effects: promoting growth at low levels, but causing inhibition or toxicity at higher concentrations with prolonged treatment. Nitrogen application enhanced the nitrogen-metabolizing enzyme activity in *M. thunbergii* leaves. The leaf NR, GS, and GOGAT activities showed initial increases, followed by declines with treatment duration, while the GDH activity exhibited sustained increases. All treatments increased the tissue nitrogen content and the nitrogen accumulation of the stems and roots, but decreased the transfer factor. While urea enhanced the foliar nitrogen accumulation, ammonium sulfate and sodium nitrate reduced it.

**Table 9:** Comparative summary of the effects of nitrogen fertilizer treatments on *Machilus thunbergii* seedlings.

Treatment Indicator		Urea	Ammonium sulfate	Sodium nitrate	Control
Growth indicator	Branch and leaf morphology	The lightest symptoms (1/4 of the seedling leaves scorching) finally	the second most severe symptoms (following the [most severe treatment group]) (1–3 seedling leaves scorching) finally	Elicited the earliest (at 40 DAT) and the most severe symptoms	Normal growth during the whole experiment
	Height growth	Second only to the control	Lower than the control and the urea treatment	Lowest growth throughout the experiment	Optimal growth throughout the experiment
	Diameter growth	Second only to the control	Lowest growth throughout the experiment, but close to the sodium nitrate treatment	Lower than the control and the urea treatment; slightly higher than the ammonium sulfate treatment	Optimal growth throughout the experiment
	Biomass	Highest total biomass; maximum biomass of all plant parts across four treatments	Total biomass is greater than that of the sodium nitrate group; leaf biomass is significantly lower than that of the control.	Lowest total biomass; leaf biomass is lower than that of the ammonium sulfate group.	Highest leaf biomass proportion; total biomass second only to the urea group
Root morphology		Best root morphology (all five root indicators)	Worse root morphology than the control and the urea treatment; with greater length, surface area, and tips than the sodium nitrate treatment	Worse root morphology than the control and the urea treatment; with greater volume and mean root basal diameter than the ammonium sulfate treatment	Lowest values in surface area, volume, and tips
Nitrogen metabolism	Enzyme activity	Three enzymes (excluding GDH) were the highest	NR and GDH activities are relatively low, while GS and GOGAT activities are relatively high	GDH activity is relatively high; GOGAT and GS activities are lower than the other two treatments; no significant difference in NR activity compared to the control	Remained basically the lowest value
	Nitrogen content	Higher than the control only	Higher than the control and the urea treatment	Highest values in leaves and stems, higher than the control and the urea treatment in roots	Lowest in all parts
	Nitrogen accumulation	Leaf: highest nitrogen accumulation; stem and root: higher than the control only	Leaf: higher than sodium nitrate only; stem: lower than sodium nitrate only; root: highest nitrogen accumulation	Leaf: lowest nitrogen accumulation; stem: highest nitrogen accumulation; root: higher than the control	Stem and root: lowest nitrogen accumulation; leaf: second only to urea treatment
	Transfer factor	Second highest	Third highest	Lowest	Highest

*DAT*, days after transplantation; *GDH*, glutamate dehydrogenase; *NR*, nitrate reductase; *GS*, glutamine synthetase; *GOGAT*, glutamate synthetase.

This study demonstrated that, among the tested nitrogen fertilizers, urea exhibited significant superiority in promoting the growth of *M. thunbergii* seedlings and in enhancing the activity of the key nitrogen metabolism enzymes, establishing it as a preferred fertilizer source for the seedling stage. Notably, the different forms of nitrogen exerted inhibitory effects of varying intensities on the seedling nitrogen transport capacity. However, the underlying mechanisms remain unclear and warrant further investigation. Furthermore, this research did not encompass changes in the content of nutritional components such as proteins, soluble sugars, and starch within the seedlings, which are closely linked to nitrogen metabolism and carbon–nitrogen balance. This study revealed synergistic and trade-off effects between organ growth and nitrogen metabolism functions. Building upon this discovery, future research should focus on the following directions to advance the conservation of *M. thunbergii* species. Firstly, a systematic evaluation of integrated carbon–nitrogen metabolic responses is needed. On the basis of existing physiological and growth indicators, simultaneous analysis of the dynamic changes in nutrients such as proteins, soluble sugars, and starch in seedlings after fertilization should be conducted, which will enable a more comprehensive assessment of the effects of nitrogen fertilizers at the level of carbon–nitrogen balance, thereby revealing the chemical basis of resource allocation and trade-offs. Secondly, an in-depth analysis of the molecular mechanisms underlying the advantages of urea is essential. Moving beyond current phenotypic observations, research should delve into the key pathways through which urea regulates efficient nitrogen metabolism in *M. thunbergii* at the molecular level, including protein activity, enzyme kinetics, and related gene expression, which will provide a theoretical basis for precision fertilization management. Finally, the regulatory principles governing resource allocation trade-offs should be elucidated. In response to the negative correlation between seedling growth and nitrogen storage observed in this study, future investigations should employ methods such as controlling source–sink relationships or applying metabolic inhibitors. This approach will allow for an in-depth study of the regulatory signals governing resource allocation and nitrogen metabolism in *M. thunbergii* seedlings under nitrogen-enriched environments, thereby refining the theory of resource allocation in environmental adaptation.

## Data Availability

The original contributions presented in the study are included in the article/**Supplementary Material**. Further inquiries can be directed to the corresponding authors.
