# Supplementary figures and images for: Effects of different nitrogen treatments on the growth and nitrogen metabolism of Machilus thunbergii seedlings
Source: Front Plant Sci. 2025 Dec 17;16:1684502. doi: 10.3389/fpls.2025.1684502 (PMC12753969; doi:10.3389/fpls.2025.1684502)

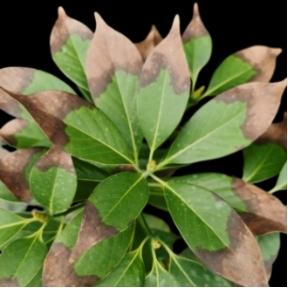

Supplement: Supplementary file 2 [file Image1.jpeg]

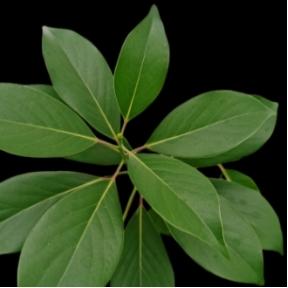

Supplement: Supplementary file 3 [file Image2.jpeg]

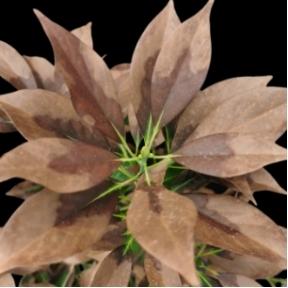

Supplement: Supplementary file 4 [file Image3.jpeg]

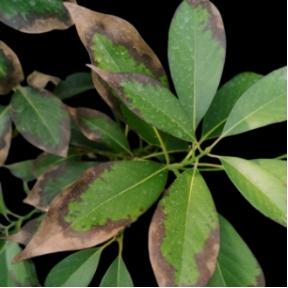

Supplement: Supplementary file 5 [file Image4.jpeg]

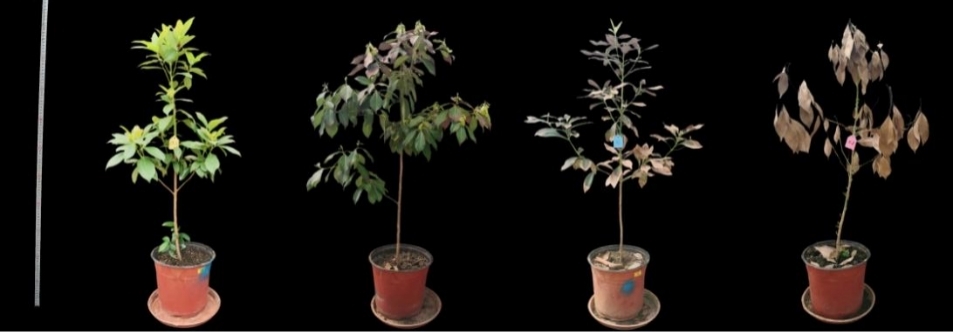

Supplement: Supplementary file 6 [file Image5.jpeg]

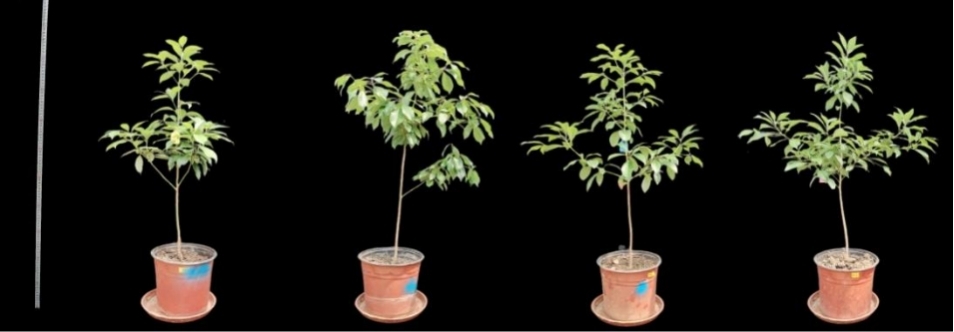

Supplement: Supplementary file 7 [file Image6.jpeg]

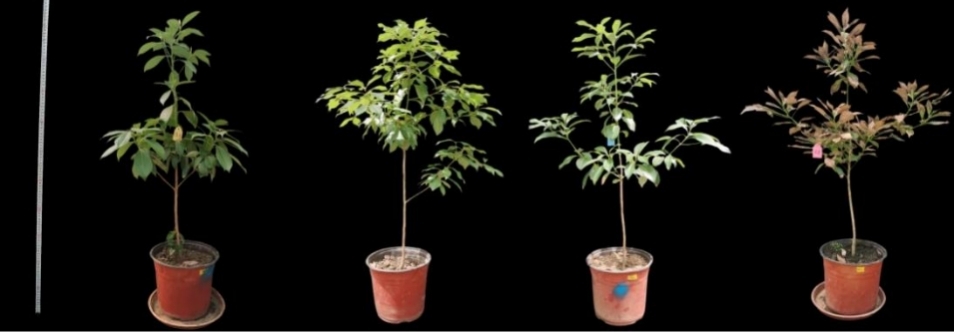

Supplement: Supplementary file 8 [file Image7.jpeg]

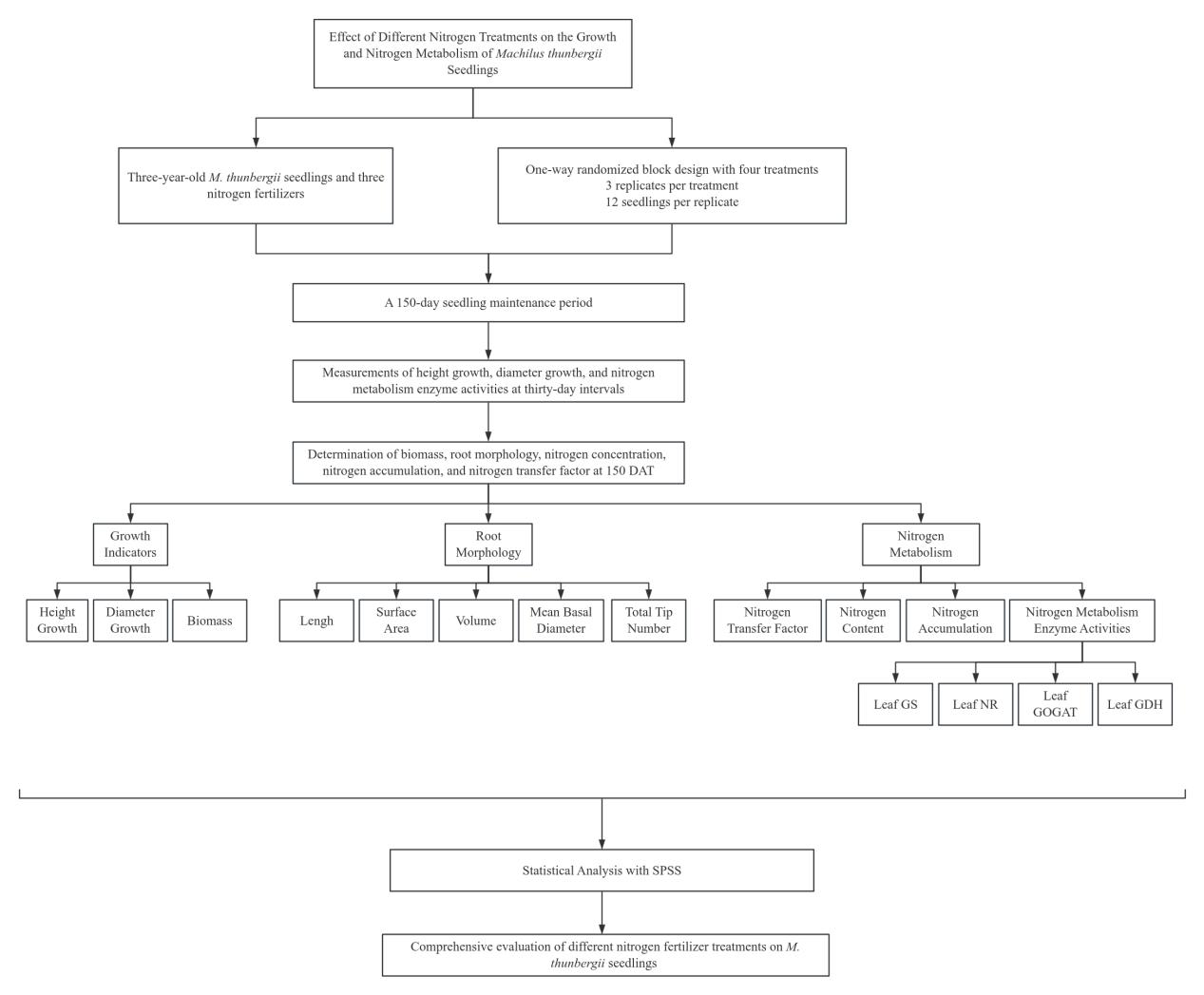

Supplement: Supplementary file 9 [file Image8.jpeg]
